# Reactive Oxygen Species-Mediated Damage of Retinal Neurons: Drug Development Targets for Therapies of Chronic Neurodegeneration of the Retina

**DOI:** 10.3390/ijms19113362

**Published:** 2018-10-27

**Authors:** Landon J. Rohowetz, Jacob G. Kraus, Peter Koulen

**Affiliations:** 1 Vision Research Center, Department of Ophthalmology, School of Medicine, University of Missouri-Kansas City, 2411 Holmes St., Kansas City, MO 64108, USA; ljrvx8@mail.umkc.edu (L.J.R.); jgkppb@umkc.edu (J.G.K.); 2 Department of Biomedical Sciences, School of Medicine, University of Missouri-Kansas City, 2411 Holmes St., Kansas City, MO 64108, USA

**Keywords:** reactive oxygen species, oxidative stress, glaucoma, diabetic retinopathy, age-related macular degeneration, biologicals, small molecules, therapy, retina

## Abstract

The significance of oxidative stress in the development of chronic neurodegenerative diseases of the retina has become increasingly apparent in recent years. Reactive oxygen species (ROS) are free radicals produced at low levels as a result of normal cellular metabolism that are ultimately metabolized and detoxified by endogenous and exogenous mechanisms. In the presence of oxidative cellular stress, ROS are produced in excess, resulting in cellular injury and death and ultimately leading to tissue and organ dysfunction. Recent studies have investigated the role of excess ROS in the pathogenesis and development of chronic neurodegenerative diseases of the retina including glaucoma, diabetic retinopathy, and age-related macular degeneration. Findings from these studies are promising insofar as they provide clear rationales for innovative treatment and prevention strategies of these prevalent and disabling diseases where currently therapeutic options are limited. Here, we briefly outline recent developments that have contributed to our understanding of the role of ROS in the pathogenesis of chronic neurodegenerative diseases of the retina. We then examine and analyze the peer-reviewed evidence in support of ROS as targets for therapy development in the area of chronic neurodegeneration of the retina.

## 1. Basic Science Evidence for a Role of Reactive Oxygen Species in Disease Development

The generation of reactive oxygen species (ROS) has recently been linked to normal physiological signaling and protective mechanisms in the retina by mediating a survival reaction when exposed to apoptotic stimuli [1,2], activating the pro-survival extracellular signal-related kinase 1/2 pathway in retinal Müller cells [3] and inducing cell survival through endoplasmic reticulum-mediated stress signaling [4]. However, this is contrasted by the widely accepted notion that ROS production can contribute to and result from several pathological processes in the retina including cellular injury during post-ischemic conditions [5], aging [6], and apoptosis [7,8], leading to an impaired redox equilibrium potentially decreasing pro-survival signaling and promoting disease progression.

## 2. Formation of Reactive Oxygen Species as a Consequence of Normal Activity of Retinal Neurons

### 2.1. Mitochondria

The high metabolic and oxidative phosphorylation rate of retinal tissue likely represents the main source of ROS generation in mitochondria of healthy retinal neurons under physiological conditions [9]. In addition, Quinlan and colleagues suggest that oxidizing glutamate results in the generation of even higher levels of superoxide within mitochondria [10], which has high relevance for ROS generation in the predominantly glutamatergic neuronal signaling pathways of the retina [11].

### 2.2. Light

The most unique source of ROS generation in the retina when compared to other tissues in the central nervous system is related to the retina’s main signaling pathway and its external stimulus, light. The retina converts visible light into electrical impulses, allowing one to perceive a pattern of photons as light and color; however, studies by Grimm et al. and Kuse et al. demonstrate that prolonged retinal excitation by light induces ROS generation and photoreceptor death [12,13]. The cause of light-induced damage by ROS appears to be related to photobleaching of rhodopsin [14] or from other retinal-specific compounds like lipofuscin or A2E [15,16] that can stimulate protein oxidation and lipid peroxidation. It is interesting to note that delayed chain branching, the joining or separating of free radicals to form other radicals may induce an emission of photons or energy transfer, potentially leading to a cycle of ROS generation by external photons, which produces free radicals that undergo delayed chain branching potentially creating more photons [17].

### 2.3. NADPH Oxidase (NOX Family)

Another physiological mode of ROS production in the retina results from the activity of nicotinamide adenine dinucleotide phosphate (NADPH) oxidase, a plasma membrane bound complex, which generates superoxide extracellularly as a defense against bacteria and fungi [18] and contributes to angiogenesis in the retinal pigment epithelium (RPE) [19] and phagocytosis [20].

### 2.4. Xanthine Oxidase

Xanthine oxidase activity contributes to the catabolism of purines at high phosphoribosyl pyrophosphate concentrations and to cellular metabolism of purines such as adenosine monophosphate, inosine monophosphate, xanthosine monophosphate, and guanosine monophosphate and ribonucleic acid (RNA)/deoxyribonucleic acid (DNA) damage [21]. Xanthine oxidase was observed in human retinas and identified in cone photoreceptors suggesting xanthine oxidase may contribute to oxidative stress, but only under conditions of nutrient starvation or ischemia/hypoxia.

## 3. Formation of Reactive Oxygen Species Resulting from Disease Processes Affecting Retinal Neurons

### 3.1. Glaucoma

Glaucoma is a chronic retinal disease associated with increased intraocular pressure (IOP) and degeneration of retinal ganglion cells (RGCs) [22,23,24,25]. Many treatment approaches have focused on reduction of IOP. However, elevated IOP does not account for all cases of glaucoma and not all individuals with elevated IOP develop signs of glaucoma [25,26]. Thus, there is a need for direct therapeutic approaches targeted elsewhere. One possible set of targets, which is associated with the pathogenesis of glaucoma [22,23,24,25,27,28], is a range of ROS. Indeed, decreasing the quantity and effect of ROS in and on RGCs through a variety of mechanisms are active areas of investigation. ROS production plays a critical role in RGC apoptosis signaling and in disrupting the antioxidant capacity of surrounding glial cells. Increased IOP levels are linked to an increase in endogenous ROS production and levels located within the trabecular meshwork (TM) [29] and abnormal mitochondria function in RGCs [30], indicating that ROS concentrations may begin to rise before the onset of glaucoma and continue to increase as RGCs begin apoptosis [30]. Additionally, direct trauma to the optic nerve also increases ROS production in RGCs [31] peaking at four days after optic nerve crush and maintaining an elevated level through seven days [31].

Calcium serves a major role in normal intracellular metabolism and signaling, often interacting with other systems including those involved in ROS regulation. However, an excess or imbalance of intracellular calcium can lead to the generation of excess ROS and metabolic derangement. Abnormal calcium influx may occur through a variety of insults, including loss of cell membrane integrity and inhibition of calcium ATPase activity. Intracellularly, calcium ions disrupt mitochondrial function, leading to increased generation of ROS [32]. Moreover, ROS themselves are known to inhibit calcium ATPase activity in neurons [33], resulting in a synergistic effect between calcium and ROS. Overstimulation of N-methyl-D-aspartate (NMDA) receptors leads to excess intracellular calcium, activation of nitric oxide (NO) synthase and overproduction of superoxide and NO, leading to eventual cell death [34].

ROS are also notorious initiators of lipid peroxidation, a process that can lead to cell membrane damage and subsequent cellular dysfunction that accompanies aging and disease. Lipid peroxidation of the mitochondrial membrane may contribute to dysfunction and apoptosis in glaucomatous RGCs [35,36]. The incorporation of antioxidants into mitochondrial membranes may prevent this process [37]. Indeed, a mitochondrial-targeted plastoquinone-containing antioxidant, 10-(6′-pastioquinonyl) decyltriphenylphosphonium (SkQ1) has been shown to reverse traits of experimental glaucoma in rabbits [35]. Likewise, choric acid and 3,5-dicaffeoylquinic acid of ethanol extract of *Crepidastrum denticulatum* have been shown to reduce lipid peroxidation and protect against RGC death in a mouse model of glaucoma [38]. Furthermore, molecular hydrogen has been demonstrated to protect lipids from peroxidation, likely via peroxynitrite scavenging, which may contribute to its ability to prevent retinal cell apoptosis [34].

As alluded to above, mitochondrial damage plays a large role in the pathogenesis of glaucoma, as mitochondria are usually the main source of ROS or principal mediators of ROS production by other organelles [39]. Mitochondrial stress leads to the release of cell death mediators, which compromise oxidative phosphorylation, leading to inefficient use of protons and the relative over- and underproduction of ROS and adenosine triphosphate (ATP) respectively [28,40]. The associated increase in ROS may be involved in the propagation of RGC apoptosis via mediation of mitochondrial permeability transition, alteration of mitochondrial membrane potential, and release of pro-apoptotic proteins [28,41]. SkQ1, mentioned previously, has been shown to prevent mitochondrial damage in fibroblasts in the presence of oxidative stress and stimulate formation of the mitochondrial network in the absence of oxidative stress by reducing endogenous ROS levels [42]. SkQ1 has also been demonstrated to prevent ROS-induced necrosis in HeLa cells [43].

Cell death mediators released in response to cell stress, including hypoxia, compromise mitochondrial oxidative phosphorylation, leading to, among other pro-death events, the production of ROS. The administration of tempol, which has been shown to prevent ROS generation and neuronal cell death, increases survival of RGCs exposed to tumor necrosis factor-alpha (TNF-α) and hypoxia in the presence of a caspase inhibitor [28]. Furthermore, crocin, a carotenoid, has been shown to suppress production of ROS, increase mitochondrial membrane potential, and enhance viability in hydrogen peroxide (H_2_O_2_)-insulted RGCs [41]. Molecular hydrogen has also been demonstrated to suppress loss of mitochondrial membrane potential and apoptosis in retinal cells, presumably via peroxynitrite scavenging [34]. Administration of γ-synuclein antibodies, which are downregulated in glaucoma patients, has been shown to increase viability, decrease ROS production, and increase the anti-apoptotic protein expression pattern in oxidatively stressed RGCs [44].

In addition to preventing the overproduction of ROS, cells regulate and manage oxidative stress by scavenging or eliminating ROS that are already formed. Much of the evidence for the protective effect of ROS scavenging in the retina comes from experiments involving the administration of the ROS scavengers themselves or exogenous substances that increase their expression and/or activity in the eye. For example, the aforementioned ethanol extract of *Crepidastrum denticulatum* has demonstrated the ability to scavenge superoxide and hydroxyl free radicals, which may contribute to its ability to reduce glaucoma-related RGC death [38]. Similarly, lignans found in *Eucommia ulmoides* extract up-regulate the activity of ROS-scavenging enzymes including superoxide dismutase (SOD), glutathione peroxidase, and catalase, which may explain the extract’s protective effect on RGCs [45]. Moreover, as a response to oxidative stress, RGCs upregulate the production of heme oxygenase (HO) and ceruloplasmin [36]. Likewise, valproic acid, a histone deacetylase inhibitor used for treatment of epilepsy and other conditions, has been shown to increase levels of SOD, glutathione peroxidase, and catalase in rat retinas exposed to ischemia/perfusion injury, while correspondingly decreasing cell death [46]. Spermidine, an endogenous ROS scavenger, has been shown to reduce oxidative stress and suppress RGC death in mouse models of glaucoma [47,48]. Furthermore, α-lipoic acid may contribute to RGC preservation via direct ROS scavenging and antioxidant gene induction [36]. Tempol, a RGC-protective compound mentioned above, has been shown to inhibit peroxynitrite, a reactive oxidant formed from NO and superoxide [28,49]. Molecular hydrogen has also been demonstrated to scavenge peroxynitrite and reduce apoptosis in retinal cells [34]. Moreover, gene therapy with SOD2 and catalase decreases RGC death in a mouse model of glaucoma (optic nerve crush) [50]. Obviously, ROS scavengers play a significant role in the protection against glaucoma and can be produced and activated through a variety of mechanisms.

The renin-angiotensin system has also been implicated in the pathogenesis of glaucoma and RGC death. In RGCs of a glaucoma mouse model, oxidative stress upregulates angiotensin II receptors (a similar effect to that seen in vascular smooth muscle cells) and toll-like receptors, leading to increased NO expression and RGC death. Accordingly, candesartan, an angiotensin II receptor antagonist has been shown to interrupt this pathway, leading to neuroprotection and increased RGC viability without altering IOP [51].

Light’s oxidizing effect on the retina may also contribute to the pathogenesis of glaucoma. Light has been demonstrated to increase ROS production and decrease viability in RGCs. The antioxidant Trolox has been shown to dampen these effects [52]. Similarly, geranylgeranylacetone, an antioxidant that has been shown to protect RGCs in mouse models of normal-tension glaucoma [53], has also been shown to decrease markers of oxidative stress caused by light in mice retinas [54].

Light also causes oxidative stress in anterior chamber tissues by inducing the formation of 8-hydroxy-2′-deoxyguanosine in DNA via singlet oxygen mechanisms. Levels of 8-hydroxy-2′-deoxyguanosine are higher in the TM of glaucomatous eyes than control eyes. The TM is the most ROS-sensitive tissue in the eye, most likely because, under normal conditions, it is not directly exposed to light and thus does not need defense mechanisms against the deleterious effects of light [55,56]. When it is exposed to ROS via circulating aqueous humor; however, there is TM cell loss and subsequent aqueous humor outflow blockage [57], which may contribute to the IOP associated with glaucoma. Outflow blockage is even more pronounced in the presence of oxidative stress when glutathione (GSH) levels are depleted in the TM [58], providing further evidence that ROS may be the source of the outflow blockage.

Glaucoma has also been associated with alterations in perfusion and vascular tone [59], and ROS have been shown to influence vascular tone [60]. Thus, increased ROS levels characteristic of glaucoma may contribute to its pathogenesis via alteration of ocular perfusion.

As alluded to previously, histone deacetylation has also been implicated as a contributor to the pathogenesis of glaucoma. Inhibition of histone deacetylation acts synergistically with neurotrophic factors in increasing RGC survival [61]. Moreover, valproic acid, a common histone deacetylase inhibitor has been demonstrated to decrease oxidative stress and cell death and increase activities of SOD, glutathione peroxidase, and catalase in rat retinas exposed to ischemia/reperfusion injury (a supposed contributor to the accumulation of ROS in glaucomatous retinas) [46]. Thus, inhibition of histone deacetylation, a commonly manipulated process in the treatment of other diseases, may be therapeutically efficacious in preventing the ROS-mediated dysfunction of glaucoma.

Dysregulation of nuclear factor erythroid-derived 2 (Nrf2), a transcription factor that is activated in the presence of increased oxidative stress, may also contribute to the pathogenesis of glaucoma. Nrf2 controls the expression of many antioxidant proteins that may protect against the progression of glaucoma. Accordingly, RGC death is increased in an Nrf2 knockout mice model of glaucoma (nerve crush). Moreover, administration of 1-(2-cyano-3-,12-dioxooleana-1,9(11)-dien-28-oyl) imidazole, an Nrf2 activator, increases expression of antioxidant enzymes and prevents RGC death [62]. Similarly, valproic acid, α-lipoic acid, and lignans from *Eucommia ulmoides* extract, all of which are neuroprotective to RGCs, increase the activity of Nrf2 [24,36,45]. Moreover, retinal gene therapy with Nrf2 decreases RGC death after nerve crush [50]. Thus, activating or suppressing the inhibition of Nrf2 activity may represent potential therapeutic approaches in glaucoma.

Other targets for glaucoma treatment include various proteins that are activated in response to oxidative stress. For example, ROS produced in response to stress activate apoptosis signal-regulating kinase 1 (ASK1) by removing thioredoxin, an inhibitor of ASK1. Activation of ASK1, in turn, leads to apoptosis. Treatment of cells with thioredoxin and N-acetylcysteine (NAC) inhibits ASK1 and thus apoptosis [63]. Furthermore, spermidine, an endogenous ROS scavenger, inhibits the activation of ASK1, reduces oxidative stress levels in the retina, and decreases RGC death in mouse models of glaucoma [47,48]. Moreover, ASK1 gene deletion results in reduced oxidative stress levels and decreased RGC death in glaucoma mouse models [64,65,66].

Similarly, overexpression of dedicator of cytokinesis 3 (Dock3), an atypical guanine exchange factor, inhibits the ASK1-p38 pathway and decreases retinal degeneration after optic nerve injury [67]. Dock3 overexpression also decreases RGC death in cell cultures exposed to H_2_O_2_ [68]. Furthermore, Dock3 has been shown to bind to NMDA receptors, decreasing their expression in a mouse model of glaucoma (glutamate/aspartate transporter knockout) [64,65,66]. Since NMDA receptor activation leads to ROS production, Dock3 may indirectly decrease oxidative stress by decreasing NMDA receptor stimulation [69,70]. Memantine, an NMDA receptor antagonist that has been shown to reduce ROS levels, has been demonstrated to be glioprotective in a glaucoma mouse model [71]. However, the mechanism of this glioprotection is unclear and may not be related to ROS. Adenosine monophosphate-activated protein kinase may also be involved in the defense against oxidative stress. This is evidenced by the fact that *Eucommia ulmoides* extract contributes to the activation of adenosine monophosphate-activated protein kinase signaling, which has been shown to play an essential role in *Eucommia ulmoides*’ neuroprotective effect in RGCs exposed to H_2_O_2_ [45].

It is apparent that ROS production and accumulation is regulated through a variety of mechanisms. Modulation of many of these individual mechanisms, including lipid peroxidation, mitochondrial damage, ROS scavenging, histone deacetylation inhibition, Nrf2 activation and various protein pathways seems to afford at least some protection against RGC death and the manifestations characteristic of glaucoma. Further investigation should seek to uncover the most potent, efficacious and acceptable methods for prevention and treatment of this debilitating condition.

### 3.2. Diabetic Retinopathy

Diabetic retinopathy (DR) is a disease characterized by retinal damage and corresponding visual defects in the setting of long-standing diabetes. DR is the most common cause of vision loss in adults 20–74 years old, and patients with diabetes have a 90% chance of developing DR within 25 years of diagnosis [25,72]. Hyperglycemia, the main driving force of DR leads to an array of metabolic and functional derangements in retinal vascular and neuronal cells [22,25,73,74,75,76]. The mechanism of these derangements is not completely understood but ROS appear to play a very significant role [27,73,74,75,76,77]. Diabetes induces the overproduction of ROS including superoxide in the retina [78]. In fact, the first cellular response to hyperglycemia in type II diabetes mellitus may be the generation of ROS. Intracellularly, ROS act directly on proteins and DNA or indirectly as second messengers modulating various signaling cascades that contribute to the pathogenesis of DR [73]. While prevention of DR can be accomplished by strict blood glucose control, this is often difficult to achieve, and most current therapeutic approaches attempt to address vascular changes. Recent evidence has shown that prevention of ROS formation significantly reduces or eliminates the end organ damage associated with the micro- and macro-vascular pathology in diabetes, including retinopathy [73]. Thus, continued investigation into the benefits of ROS suppression in DR is warranted.

Retinal oxygen consumption and blood flow decreases under diabetic conditions [79,80] explaining why the retina undergoes hypoxia and the stimulation of angiogenesis, metabolism [81], and ROS production [82,83]. Obrosova and colleagues [81] demonstrated that retinal metabolism is increased in streptozotocin-induced diabetes, and with increased metabolism comes an increase in ROS production from the mitochondria, where superoxide is the main reactive species generated with minor contributions from NADPH oxidase and light [81]. As DR progresses, retinal swelling and hemorrhages and disruption of photoreceptor signaling are accompanied by an increased generation of ROS in photoreceptors [84]. Increased metabolic rates and light-induced ROS generation further contribute gradually to overall higher ROS levels especially after additional damage to the mitochondria occurs [84]. During maladaptive neovascularization, retinal Nox family isoforms are activated, further increasing ROS levels in the cytoplasm and inducing mitochondrial membrane damage [85]. Furthermore, Choudhuri et al. [86] suggest that under continuous oxidative stress, advanced glycation end products (AGEs) might mediate the progression of non-proliferative retinopathy to the proliferative state [86].

Previous reviews have illustrated some of the benefits of antioxidant therapy on DR. For example, Payne et al. outlined the effects of ROS on retinal neurons and argued that new therapy should target both antioxidation and neuroprotection [24]. Other reviews have illustrated major pathways stimulated by hyperglycemia, including the polyol pathway, hexosamine pathway, protein kinase C (PKC) activation, and formation of AGEs, and how they relate to oxidative stress [73,74,75,76]. Furthermore, Mitra, Conley, and Naash have provided evidence for a nanotechnological approach to treating DR [27]. In this review, we will further illustrate the role of ROS on retinal neurodegeneration in DR and examine the potential of ROS as therapeutic targets.

AGEs are carbohydrate posttranslational protein modifications in age and diabetes that activate receptor for AGEs (RAGEs) to initiate an inflammatory response [25]. They are significant contributors to microvascular damage in diabetes mellitus and correlate with the degree of DR. It is likely that ROS contribute to dysfunction caused by AGEs. Indeed, AGEs increase production of ROS, and olmesartan, an angiotensin II receptor antagonist, inhibits AGE-potentiated inflammatory reactions by suppressing ROS formation [87]. It is also believed that ROS contribute to RAGE protein overexpression through activation of NF-κB [88]. Pigment epithelium-derived growth factor (PEDF), a multifunctional glycoprotein with neuronal differentiating activity, has been shown to decrease RAGE messenger RNA (mRNA) levels in rat eyes, block AGE-induced RAGE gene induction in microvascular endothelial cells (NAC also showed this effect), and completely inhibit superoxide generation and NF-κB activation in AGE-exposed endothelial cells [89]. Further investigation should seek to elucidate the effectiveness of preventing the AGE-induced ROS-mediated effects associated with DR. It seems that a variety of processes can be targeted, including NF-κB activation, RAGE production, and direct ROS scavenging.

The phosphoinositide 3-kinase (PI3K)/protein kinase B (Akt) pathway may also be implicated in the ROS-mediated pathogenesis of DR. For example, insulin-like growth factor-1 is an endogenous growth factor proposed to act through the PI3K/Akt pathway to inhibit RPE cell death to reduce/delay the development of DR. Exogenous administration of insulin-like growth factor-1 in a model of NO toxicity characteristic of some patients with DR prevents RPE cell death, likely through activation of the PI3K/Akt pathway [90]. Moreover, vascular endothelial growth factor (VEGF)-A may promote RPE cell survival through the Akt pathway in the presence of oxidative stress [91]. On the other hand, insulin has been reported to increase ROS production and induce VEGF expression through the PI3K/Akt pathway in retinal endothelial cells. Moreover, Nox4-derived ROS have been suggested to regulate insulin-induced hypoxia-inducible factor (HIF)-1α and VEGF expression through the PI3K/Akt pathway [92]. As a stimulator of angiogenesis and an RPE cell survival factor, VEGF has both detrimental and beneficial effects in the retina [91,93,94,95,96,97]. Thus, in light of the close association with VEGF, further investigation is necessary to more clearly understand the consequences of PI3K/Akt pathway modulation in DR.

Over-activation of PKC, an important mediator of various enzymes, receptor pathways, and transcription factors located downstream of PI3K, is implicated in the pathogenesis of DR. PKC, over-activated by hyperglycemia-induced diacylglycerol synthesis in DR, has been shown to contribute to retinal leakage, ischemia, capillary pericyte death and neovascularization. PKC’s effects arise in part due to its stimulatory effect on NADPH oxidase and the corresponding increase in ROS. PKC-B inhibitors, particularly ruboxistaurin, are currently being investigated as possible therapeutic options for at least some of the vascular manifestations of DR. Ruboxistaurin has been shown to normalize retinal blood flow, decrease vessel permeability and neovascularization, and reduce vision loss over 3 years in patients with moderately severe to very severe non-proliferative DR [98,99]. However, ruboxistaurin does not prevent DR progression [100] and has not yet been approved for treatment of DR in the U.S. In light of these findings, further investigation is needed to determine the utility of PKC inhibitors in DR treatment.

Lipid metabolism and palmitoylation may also be involved in the pathogenesis of DR. Lipotoxic and glucolipotoxic insults increase ROS (and interleukin (IL)-1β) production via Nox2-Rac1 signaling and accelerate mitochondrial damage, exacerbating the development of DR [101]. Palmitate specifically has been shown to increase ROS generation, inhibit DNA synthesis, and induce apoptosis in endothelial cells [102]. It is suggested that, since palmitate levels are higher in the serum of diabetic patients, ROS overproduction may occur as a result of increased mitochondrial fatty acid oxidation in endothelial cells [88]. Palmitoylation of Rac1 is an important signaling event in the activation of the Tiam1-Rac1-Nox2 signaling module that produces ROS in early stages of DR. Addition of 2-bromopalmitate, a specific inhibitor of protein palmitoylation, abolishes glucose-induced Nox2 activity and ROS levels [102]. Palmitate was also found to upregulate RAGE mRNA expression in pericytes, possibly by increasing activation of ROS-activated NF-κB [88]. Beyond the circulation, it has been shown that prolonged exposure of RGCs to palmitate induces ROS overproduction (directly and via endoplasmic reticulum stress) and RGC apoptosis, a characteristic of early stage DR. Crude saponins of *P. notoginseng* have been shown to inhibit this palmitate-induced ROS generation and apoptosis in RGCs [103]. Administration of NAC, an intracellular radical scavenger, completely prevents DNA synthesis inhibition and apoptosis in the presence of palmitate. Taken together, these findings suggest that palmitate’s harmful effects are directly mediated by generation of ROS [88]. Thus, suppressing the action of palmitate and its downstream ROS-mediated processes may protect the retina against dysfunction associated with DR.

The severity of retinopathy is directly correlated with triglyceride level and inversely correlated with high-density lipoprotein levels [101]. Oxidized low-density lipoprotein (LDL) is present in human retinas proportionate to DR severity, and is even present in the absence of clinically apparent DR. Oxidized LDL is absent in normal control human retinas [104]. IgG is also present in human DR retinas, which may contribute to the formation of oxidized LDL immune complexes, which enhance inflammatory cytokine secretion and trigger apoptosis via oxidative stress to a greater degree than oxidized LDL [104]. This immune complex formation represents an extravascular event that could theoretically be prevented through a variety of mechanisms, including antioxidant, anti-inflammatory, and immunosuppressive treatments.

As mentioned above, physiologic mechanisms exist to eliminate or reduce the impact of existing sources of oxidative stress. SOD is a major antioxidant involved in the protection against oxidative stress characteristic of DR, including oxidative stress induced by hypoxia. Hypoxia induced by cobalt chloride (CoCl_2_) produces an increase in the production of ROS and a decrease in the production of extracellular-SOD (the major SOD isozyme in blood vessel walls), leading to retinal pericyte apoptosis. Furthermore, treatment with NAC, an intracellular radical scavenger discussed above, and 4-phenyl butyric acid, an extracellular-SOD enhancer, suppresses these effects and the hypoxia-induced manifestations of DR. Thus, hypoxia may exert its pro-apoptotic effects by decreasing the production of superoxide scavengers, resulting in increased ROS accumulation [105]. On the other hand, while SOD seems to be decreased in diabetes, catalase has been shown to be elevated, suggesting that H_2_O_2_ may be the predominant source of oxidative stress in diabetes [106]. Moreover, lipoic acid has also been demonstrated to improve mitochondrial function in diabetes by scavenging superoxide radicals, possibly accounting for the fact that long-term administration prevents the development of DR in diabetic rats [107]. Furthermore, in high-glucose-treated red blood cells, pyridoxine (vitamin B6) and pyridoxamine scavenge superoxide radicals, prevent lipid peroxidation and hemoglobin glycosylation, and stimulate Na^+^/K^+^ ATPase activity [108]. Cerium oxide nanoparticles scavenge ROS by acting like superoxide dismutase and catalase, and have been shown to inhibit ROS, VEGF, and intraretinal and subretinal neovascular lesions in very low density lipoprotein receptor knockout mice. Furthermore, cerium oxide nanoparticles have been shown to promote the regression of existing vascular lesions in older mice, further demonstrating its possibility as a therapeutic agent to treat and prevent DR [109]. Leaves and extract of *Nelumbo nucifera* also have radical scavenging, ROS-inhibiting, and anti-diabetic effects (including inhibition of aldose reductase and AGE formation) [110]. Thus, as evidenced, ROS scavenging seems to have therapeutic potential in the treatment and prevention of DR through a variety of mechanisms and is worthy of further investigation as a treatment modality in DR.

The polyol pathway is suspected to play a significant role in the various complications associated with diabetes. At high glucose levels, there is excessive cellular glucose uptake, leading to increased activation of aldose reductase and the accumulation of sorbitol. Sorbitol is then oxidized by sorbitol dehydrogenase into fructose. The depletion of NADPH as a result of the reduction of glucose into sorbitol, and the accumulation of reduced nicotinamide adenine dinucleotide (NADH) as a result of sorbitol oxidation into fructose may increase oxidative stress and ROS production in diabetes [110]. NADH is a substrate for ROS production by NADH oxidase and NADPH is a cofactor for the production of GSH, an important ROS scavenger. Indeed, Amano et al. showed that conversion of sorbitol to fructose by sorbitol dehydrogenase results in ROS generation that potentiates glucose toxicity to retinal pericytes. Furthermore, NAC completely prevented this ROS generation and glucose toxicity. Fidarestat, an aldose reductase inhibitor, had the same effects, in addition to preventing sorbitol accumulation in pericytes [111]. Also, as mentioned above, the radical scavenger *Nelumbo nucifera* inhibits AR, which may suppress the production of ROS associated with the polyol pathway [110], thus preventing the complications of diabetes. Taken together, these findings indicate that the polyol pathway may be a worthy target in preventing the ROS-associated complications of DR.

Calcium dysregulation may also be involved in the pathogenesis of DR. High glucose has been shown to increase intracellular and extracellular calcium concentration. Increased intracellular calcium concentration leads to activation of nuclear factor of activated T cells (NFAT), which is involved in cytokine regulation, in addition to other processes. Furthermore, diabetes reduces the level of the anti-inflammatory cytokine IL-10. IL-10 has been suggested to limit oxidative stress and vascular dysfunction in mouse carotid arteries. Diabetic mice manifest increased NFAT-dependent transcriptional activity in retinal vessels and decreased IL-10 levels. NFAT antagonist administration has been shown to restore IL-10 to normal levels and abrogate vascular permeability in diabetic mice [112]. Thus, the role of calcium dysregulation in DR should continue to be investigated.

Hypoxia and redox state dysregulation are also believed to play significant roles in the pathogenesis of DR. Cervellati et al. have shown that CoCl_2_-induced hypoxia increases HIF-1α accumulation, ROS production, lipid peroxidation, NF-κB production, and VEGF induction in parallel to an increase in caspase 3, poly (ADP-ribose) polymerase, and consequent apoptosis in human RPEs [113]. As mentioned previously, CoCl_2_-induced hypoxia also increases ROS production and decreases extracellular-SOD production, leading to retinal pericyte apoptosis. These effects were inhibited with the administration of the antioxidant NAC [105]. Administration of vitamin C and vitamin E decreases lipid peroxidation products in CoCl_2_-induced hypoxia [113]. Thus, hypoxia and its downstream effects, particularly ROS production, may contribute to the angiogenesis and neurodegeneration characteristic of DR and may represent targets for therapy.

As a potent stimulator of angiogenesis, VEGF plays a critical role in the manifestation and development of DR. VEGF upregulation is a significant contributor to blood-retinal barrier (BRB) breakdown in DR [100]. As mentioned previously, CoCl_2_-induced hypoxia leads to increased ROS production and induction of VEGF and angiogenesis [113,114]. ROS in high-glucose-exposed pericytes up-regulates VEGF and is inhibited by NAC administration [111]. As alluded to previously, insulin induces Nox4-derived ROS, which are essential for HIF-1α-dependent VEGF expression [92]. Sun et al. demonstrated that intermittent glucose fluctuations enhance cell proliferation and VEGF overexpression through ROS. The antioxidants Mn(III)tetra-kis(4-benzoic acid) porphyrin chloride and thenoyltri-fluoroacetone can prevent cell proliferation, overproduction of mitochondrial ROS, and oxidative damage in human retinal endothelial cells [77]. Epigallocatechin-3-gallate, an antioxidant polyphenol of green tea leaves, has been demonstrated to protect RPE cells against death, prevent BRB remodeling, and decrease mRNA expression of angiogenic factors including matrix metalloproteinase (MMP)-9, VEGF, and VEGF Receptor-2 by inhibiting production of ROS [115].

MMPs also play a significant role in the pathogenesis of DR, particularly in ECM remodeling involved with angiogenesis. MMPs, which are controlled at least in part by ROS, are increased in diabetic patients and animal models of DR and are thought to contribute to disruption of tight junction complexes, vascular permeability and BRB dysfunction [116,117]. As mentioned above, inhibition of ROS production decreases mRNA expression and protein production of angiogenic factors including MMP-9 in RPE cells, thus preventing cell death and BRB remodeling [115]. Superoxide scavengers have been shown to inhibit glucose-induced activation of retinal capillary MMPs. Furthermore, MMP inhibitors have been shown to decrease associated apoptosis [116]. Moreover, inhibition of MMPs suppresses glucose-induced mitochondrial superoxide production and inhibits capillary cell apoptosis, suggesting that MMPs have a role in activating apoptosis of retinal capillary cells through mitochondrial dysfunction, ROS production, and a corresponding increase in membrane permeability [118]. Taken together, these results suggest that ROS and MMPs may exert reciprocal/synergistic effects on each other in DR. Thus, control and regulation of either MMPs or ROS may ultimately decrease oxidative stress and the dysfunction caused by MMP over-activation.

The processes associated with heme degradation may also play a role in the pathogenesis of DR. HO-1, an enzyme that catalyzes the breakdown of heme, has demonstrated antioxidant vascular-protective properties. Likewise, HO-derived carbon monoxide prevents apoptosis and bilirubin and biliverdin have strong antioxidant properties. HO-1 is expressed by many cells in the retina at baseline but is upregulated in diabetes and in states of increased oxidative stress. Inhibition of HO enhances ROS production and the toxic effects of oxidative stress while overexpression of HO-1 prevents these toxic effects. Furthermore, HO-1 prevents the cytotoxic effects of high glucose, although the protection is transient [78]. Moreover, Metanx, a combination of the active components of vitamins B_6_, B_9_, and B_12_ has been demonstrated to upregulate HO-1 and reduce diabetes-induced retinal superoxide generation. However, Metanx has not been shown to inhibit degeneration of retinal capillaries or capillary pericytes, perhaps because more complete elimination of ROS was not achieved in the associated study [119].

NADPH oxidase, an enzyme that produces superoxide is suggested to play a significant role in DR. PKC activation and the AGE-RAGE interaction discussed above lead to the generation of ROS via NADPH oxidase [120,121]. The catalytic subunit, Nox2, may be particularly important, as it has been associated with diabetes-induced increases in retinal ROS, vascular permeability, and VEGF expression. Inhibition and gene deletion of Nox2 prevents the BRB breakdown, intercellular adhesion molecule 1 (ICAM-1) expression, and leukostasis characteristic of DR [122]. Nox4 knockdown also reduces insulin-stimulated ROS production [92]. Rac1-dependent NADPH oxidase-mediated ROS production is an important contributor to VEGF’s angiogenic effects. These effects may be suppressed by Rac1 inhibition [123]. Furthermore, C-reactive protein may promote the progression of DR by inhibiting retinal arteriole dilation by NADPH oxidase-mediated ROS production [124]. PEDF has been demonstrated to suppress NADPH oxidase-mediated ROS generation, leading to a reduction in IL-6 overexpression in human umbilical vein endothelial cells [125]. Thus, downregulation of NADPH oxidase seems to be an efficient method for decreasing ROS production in DR and is deserving of further investigation.

Interleukins are proposed to act in conjunction with ROS to contribute to the pathogenesis of DR. IL-1β is increased in retinal endothelial cells in the presence of glucolipotoxicity and has a suggested role in the BRB breakdown and oxidative stress characteristic of DR [101,125,126]. Tetramethylpyrazine, a component of the herb *Ligusticum chuanxiong*, prevents IL-1β-mediated cell damage, likely, at least in part, through inhibition of ROS generation [126]. As discussed above, PEDF also reduces IL-6 production in human umbilical vein endothelial cells, at least partially through the reduction of ROS. IL-1 and IL-6 both are suggested to contribute to atherosclerosis, with the former (along with other cytokines) likely stimulating production of the latter in endothelial cells [125]. Further investigation is needed to understand the role of interleukins in DR.

The visual cycle may also contribute to ROS production induced by diabetes and its modulation may have therapeutic benefits in DR. Ret-NH_2_ (retinylamine) inhibits RPE65, an enzyme involved in the visual cycle, and has been shown to reduce diabetes-induced effects related to oxidative stress. It is postulated to do so by slowing the visual cycle and moderating corresponding phototransduction and mitochondrial demand, which reduces ROS production and decreases subsequent capillary degeneration [127].

Modulation of Nrf2 activity may also be a potential therapeutic option for DR. In times of stress, Nrf2 dissociates from its cytosolic inhibitor, Kelch-like ECH-associated protein 1, and translocates to the nucleus to stimulate transcription of antioxidant genes. Diabetes increases retinal Nrf2 binding with Kelch-like ECH-associated protein 1 and decreases its DNA-binding ability, leading to decreased antioxidant gene transcription. An Nrf2 inducer and Kelch-like ECH-associated protein 1-small interfering RNA (siRNA) prevent these effects [128].

Furthermore, the WNT pathway has been shown to be activated in DR and may be a target for therapy. ROS-induced lipid peroxidation products have been shown to be activators of the canonical WNT pathway, implicating ROS production and these corresponding downstream processes as possible targets for DR treatment [129].

NO synthase uncoupling may also be involved in DR, as evidenced by the fact that diabetes is associated with upregulation of arginase I expression, which decreases bioavailable NO and correspondingly increases superoxide formation by NO synthase [130].

Various other compounds may be implicated in the pathogenesis and treatment of DR. Lipoic acid has been shown to abrogate mitochondrial dysfunction, increase GSH levels, inhibit NF-κB, and prevent apoptosis of retinal capillary cells and the development of DR in diabetic rats [131,132,133,134]. Estrogen may also be involved in retinal protection in DR, as it has been shown that 17β-estradiol reduces ROS levels and decreases apoptosis of RGCs in a high-glucose environment [135]. As alluded to previously, TNF-α increases ROS levels in models of DR and thus acts as another potential therapeutic target [125]. Overexpression of uncoupling protein 2, a mitochondrial modulator that has protective effects against endothelial dysfunction, has also been demonstrated to inhibit apoptosis and decrease ROS levels in human umbilical vein endothelial cells [136].

### 3.3. Age-Related Macular Degeneration

Age-related macular degeneration (AMD) is a disease characterized by retinal deposits, lipofuscin deposition, loss of RPE, and choroidal neovascularization (CNV; wet form) and is a common cause of blindness with aging. Cumulative oxidative stress is the presumed mechanism leading to neurodegeneration seen in AMD [22,25]. RPE, specialized epithelium between the neural retina and Bruch’s membrane that maintains homeostasis between neurons and the choroid, is prone to oxidative injury due to constant light exposure, increased metabolic activity, and accumulation of oxidized lipoproteins [137]. Previous work has demonstrated the effects of ROS in increasing mitochondrial dysfunction, generation of drusen, inflammation, angiogenesis, vascular damage, production of AGEs, and RPE cell death with a corresponding decrease in B-cell lymphoma 2 (Bcl-2) expression in AMD. Proposed contributors to ROS production and accumulation in AMD include light, diet, cigarette smoking, photosensitizers (i.e., lipofuscin) and cardiovascular disease [23,93,137,138,139]. RPE cells of AMD patients have been shown to lose antioxidant activity with time, resulting in increased oxidative stress and alteration of the surrounding environment, causing photoreceptor cell degeneration [139,140].

The contribution of ROS to AMD disease development is still largely unknown, but the antioxidant capacity of retinal cells decreases with age as seen in other tissues allowing for a rise of oxidative conditions that can irreversibly damage photoreceptor and RPE cells and result in apoptosis [141,142]. Also, abnormal activity of and damage to mitochondria contributes to elevated levels of ROS production that result from changes in mitochondria number, size and integrity with age [143]. As retinal mitochondria age, modulation of their activity occurs causing a decrease in their SOD enzymatic antioxidant activity and with the high oxygen consumption rate of the photoreceptors, RPE cells are unable to scrub superoxide to add to increasing oxidative stress in AMD [143]. Retinal apoptosis can result from the formation of drusen and lipofuscin where the aggregation of these granules restricts blood and nutrient flow to the outer retina and generates multiple ROS, including superoxide when exposed to blue light [13,144,145,146] as blue light has also been linked to ROS production by affecting oxidative phosphorylation outside mitochondria in the outer segments of rod photoreceptors [147].

SOD, an ROS scavenger discussed previously, plays a major role in defense against AMD manifestations induced by oxidative stress. Serotonin receptor agonists, multivitamins, resveratrol, curcumin, NAC, lutein, zeaxanthin, low-dose irradiation, metal oxide nanoparticles, and polyphenol compounds have all also been implicated as protective agents against oxidative stress-induced AMD [22,25,27,148]. Rapamycin, an immunosuppressant and anti-inflammatory may be effective in treating wet AMD [25]. We will illustrate the most recent findings and outline the possible approaches to targeting ROS and oxidative stress for the treatment of AMD.

The role of ROS scavenging in the treatment of AMD has been an area of much investigation. It has been shown that SOD mutations promote neovascularization, which is reduced by antioxidants [149]. Diarylheptanoids isolated from *Curcuma comosa*, which have similar structures as curcumin, have been shown to attenuate the H_2_O_2_-induced decrease of glutathione peroxidase and SOD activities, increase catalase activity, and decrease RPE apoptosis [150]. Furthermore, 15-deoxy-Δ^12,14^-prostaglandin J_2_ has been shown to upregulate GSH synthesis and protect RPE cells from oxidative injury. GSH depletion reverses this protective effect and sensitizes cells to oxidative stress [151]. Resveratrol, a red wine polyphenol mentioned above, has been shown to inhibit basal and H_2_O_2_-induced oxidation and RPE cell death and hyper-proliferation via inhibition of the mitogen-activated protein kinase (MAPK)/ERK1/2 cascade [152], as well as to attenuate ROS production from altered oxidative phosphorylation outside mitochondria in the outer segments of rod photoreceptors [153]. Moreover, clotrimazole, an antifungal with free radical scavenging abilities, has been shown to protect RPE cells against oxidative stress via inhibition of ROS generation and caspase activity [154]. Alpha-mangostin, a xanthone purified from mangosteen, can also suppress retinal cell apoptosis through ROS scavenging and strengthening of the endogenous defense system via upregulation of Nrf2 activity and expression of HO-1 [155]. Pramipexole, a dopamine receptor agonist, may protect against light-induced retinal damage by increasing ROS scavenging activity and decreasing caspase activity [156]. Fullerenol, a polyhydroxylated fullerene derivative, protects RPE cells through ROS scavenging and activation of sirtuin 1, leading to a decrease in the levels of ROS-mediated p53 and p21 levels [157]. Injection of cerum oxide nanoparticles (nanoceria) has scavenging abilities by mimicking the activities of SOD and catalase and has been shown to inhibit production of ROS, suppress levels of VEGF, and promote prevention and regression of retinal neovascular lesions in a mouse model of AMD [109]. Lutein, a xanthophyll extract with antioxidant and radical scavenging abilities, has been shown to protect against AMD [158]. Clearly, ROS scavenging through a variety of mechanisms has the potential for therapeutic efficacy in AMD.

As CNV is a characteristic of AMD, VEGF is often implicated as a possible therapeutic target. Rac1, a subunit of NADPH oxidase, activation is a critical step in AMD-associated CNV. In human choroidal endothelial cells, VEGF induces activation of Rac1 and NADPH oxidase and a corresponding increase in ROS production [19,123]. ROS then induce transcription factors and genes involved in angiogenesis [123]. NAC, apocynin and diphenyleneiodonium (NADPH oxidase inhibitors), and p47 (a subunit of NADPH oxidase) knockout prevent the generation of ROS [19]. Epigallocatechin-3-gallate has also been shown to decrease mRNA expression of VEGF and VEGF receptor by inhibiting the generation of ROS [115]. Hyperglycemia may also contribute to the pathogenesis of AMD by increasing oxidative stress, which activates signal transducer and activator of transcription 3, promotes production of VEGF, and leads to CNV. Treatment with NAC rescues the severity of this CNV [159]. In light of this evidence, VEGF and its upstream activators are worthy targets for continued investigation. It should be noted that, although VEGF may contribute to the vascular pathology associated with AMD and DR, it is also a protective growth factor for retinal endothelial cells and cells of the developing and mature retina and its secretion is triggered by oxidative stress [93].

As indicated above, light is implicated as a major cause of AMD. Ultraviolet light in the retina is absorbed by proteins and DNA and may lead to the production of ROS [138,160,161]. Resveratrol has been demonstrated to suppress ultraviolet A-induced H_2_O_2_ production, lower p38 and extracellular signal-regulated kinase activation, and increase cell viability in RPE cells [160]. Lutein has been shown to decrease ultraviolet B-induced ROS production and lipid peroxidation, and subsequently reduce RPE apoptosis and increase cell viability and antioxidant enzyme activity. Lutein’s beneficial effects were enhanced in the presence of a vitamin E analog [161]. Further investigation into preventing the detrimental effects of ultraviolet light associated with AMD is warranted.

Like the previously discussed neurodegenerative diseases of the retina, Rac1/NADPH oxidase is associated with the production of ROS in AMD. Bright light exposure in a mouse model of Stargardt disease, a severe juvenile form of AMD, induces ROS production via NADPH oxidase through G-protein-coupled receptor and phospholipase C/inositol triphosphate/calcium signaling. Photoreceptor degeneration associated with this phenotype was reversed by inhibition of G_q_ or G_s_ or stimulation of G_i_ [138]. As mentioned above, NAC and p47 knockout prevent the production of ROS mediated by VEGF-stimulated NADPH oxidase in human choroidal endothelial cells [19]. Furthermore, an arylidene-thiazolidinedione without peroxisome proliferator-activator receptor-γ activity, may inhibit retinal neovascularization by scavenging VEGF-produced ROS [123].

Calcium dysregulation is also associated with oxidative stress characteristic of AMD. For example, H_2_O_2_-induced calcium mobilization in human RPE cells increases the activation of NF-κB, which subsequently activates IL-8, a pro-inflammatory cytokine believed to contribute to the pathogenesis of AMD [162]. Complement activation has also been implicated in AMD, and RPE cells may become sensitized in the presence of oxidative stress-induced intracellular ROS production. Although acute calcium influx is associated with this complement-mediated cell death, increased extracellular and intracellular calcium levels have been suggested to afford RPE cells with protection against complemented-mediated death [163]. Thus, calcium regulation and calcium’s effects in AMD are complex and context-dependent and thus require further investigation.

α-Crystallins, members of the heat shock protein family, and associated activation of the PI3K/Akt pathway may be implicated in endogenous protection against AMD. αA- and αB-crystallins are implicated as important antioxidant factors and have been demonstrated to increase in the presence of oxidative stress [139,164]. When compared with control mice, RPE cell degeneration is more severe, and ROS accumulation is increased in αA- and αB-crystallin knockout mice in the presence of oxidative stress [164,165]. Individuals whose level of αA-crystallin is decreased (e.g., cataract patients) are more susceptible to develop geographical atrophy in the presence of oxidative stress [164]. Moreover, RPE cells that have been transfected with αB-crystallin are more resistant to oxidative stress-induced cellular injury when compared with normal RPE cells [139]. This protective effect is due at least in part to upregulation of Akt phosphorylation and peroxisome proliferator-activator receptor-γ expression [165]. Likewise, astaxanthin, a carotenoid, has been shown to decrease H_2_O_2_-induced cell viability loss, apoptosis, and generation of ROS in RPE cells at least in part through activation of the Nrf-antioxidant-response element and PI3K/Akt pathways [166].

As alluded to above, regulation of gene expression may play a central role in the defense against AMD. Oxidative stress has been shown to induce the expression of phase II enzymes, including NAD(P)H quinine oxidoreductase 1, HO-1, glutamate-cysteine ligase modifier subunit, and glutamate-cysteine ligase catalytic subunit [166]. Oxidative stress also regulates the expression of activator protein 1 transcription factor genes, including *Fos* and *ATF3*, which control cell proliferation and apoptosis. Oxidative stress-induced *Fos* and *ATF3* gene expression is reduced in RPE with ascorbate supplementation, which may account for its pro-survival effect [167]. 17β-estradiol may also prevent H_2_O_2_-induced RPE cell apoptosis by regulating expression of genes associated with apoptosis [168]. As discussed above, alpha-mangostin enhances Nrf2 transcriptional activity and expression of HO-1 [154]. Furthermore, administration of sulforaphane alters the transcriptional levels of 69 genes in human RPE cells, upregulating antioxidant-related genes and downregulating inflammatory genes to consequently inhibit apoptosis [140]. Thus, gene modulation through a variety of methods should be considered as possible treatment for AMD.

Redox-active iron catalyzes the formation of lipofuscin in RPE cells. The production of lipofuscin is dependent on the intra-lysosomal formation of hydroxyl radicals via the Fenton reaction and leads to liposomal membrane peroxidation and apoptosis. Thus, iron-binding proteins may play a protective role in reducing oxidative stress associated with AMD. Indeed, cultured immortalized human RPE cells have been shown to contain significant amounts of iron-binding proteins. Furthermore, downregulation of iron-binding proteins in RPE cells makes them more susceptible to oxidative stress [169].

MMPs may also contribute to the development of AMD. Epigallocatechin-3-gallate, an antioxidant polyphenol of green tea leaves discussed earlier, limits upregulation of ROS-induced pro-angiogenic factors, including MMP-9 [115].

Uncoupling protein 2 may also be involved in protection against AMD-related ROS production. Stanniocalcin-1 prevents photoreceptor cell death, upregulates uncoupling protein 2, and decreases levels of protein adducts generated by ROS in mouse models of photoreceptor degeneration [170].

MAPK modulation may also be implicated in the pathogenesis of AMD. MAPK activation is essential to the cytoprotective effects of 15-deoxy-Δ^12,14^-prostaglandin J_2_, a cyclopentenone discussed above [151]. Moreover, artemisinin, an anti-malarial medication with ROS-reducing effects, has been shown to have anti-apoptotic properties in RPE cells through activation of the MAPK pathway (extracellular signal-regulated kinase activation) [171]. Furthermore, resveratrol reduces RPE cell hyperproliferation by inhibiting the MAPK cascade [152]. Thus, the MAPK cascade may be a therapeutic target for the prevention of AMD.

NF-κB, a transcription factor discussed earlier involved in many inflammatory and immune pathways, is also implicated in the development of AMD. Oxidative stress causes perinuclear NF-κB accumulation, mitochondrial membrane depolarization, and corresponding cell death in RPEs. Overexpression of thioredoxins, intracellular ROS scavengers, inhibits all of these effects [172]. IL-8, a cytokine activated by NF-κB, is increased in AMD due to increased levels of oxidative stress [162].

A variety of other targets have been investigated in attempts to minimize the effects of ROS and oxidative stress on the retina in AMD. Artemisinin, lutein, zeaxanthin, and β-carotene have demonstrated the ability to preserve mitochondrial membrane potential in RPE cells [171]. Zeaxanthin and lutein have also been shown to protect photoreceptors from oxidative stress-induced apoptosis and enhance photoreceptor differentiation [173]. Moreover, erythropoietin can prevent ROS-induced RPE cell apoptosis even after radical exposure, although the cellular mechanism is not clear and may not involve simple scavenging [174].

## 4. Drug Development Efforts Targeting ROS

As outlined above, the source and effects of ROS in the retina are diverse. As ROS underlie the pathology of many of the manifestations of the aforementioned chronic neurodegenerative diseases, development and implementation of therapies that afford protection against ROS will be important for prevention and treatment of these diseases. The remainder of this paper will outline current approaches to the ROS-targeted treatment of these disorders. In this section, small molecule therapy will be discussed.

### 4.1. Drug Development Efforts Targeting ROS: Signaling Pathway Activated by Reactive Oxygen Species as Indirect Targets for Drug Development

Oxidative stress triggers cellular apoptosis signaling including Jun NH_2_-terminal kinase (JNK)/stress-activated protein kinase, p38, and ASK1, as well as AGE pathways along with the “pro”-survival NF-κB pathways [175,176]. The mentioned retinal diseases commonly experience a dysregulation of mitochondria function and generate ROS through damaged mitochondrial DNA and/or an elevated metabolic rate, which leads to decreased glyceraldehyde 3-phosphate dehydrogenase activity, abnormally high activity of the AGE/RAGE, polyol, and PKC pathways alongside heightened NADPH activity [177]. In glaucoma models, an increase in ROS concentrations has been discussed as a major contributor to RGC apoptosis that works with the impaired drainage flow of aqueous humor fluid through the TM to activate the NF-κB pathway [178]. Li et al. and Liton et al. provided evidence that prolonged activation of NF-κB family factors can induce a multitude of leukocyte and interleukin signaling molecules such as endothelial-leukocyte adhesion molecule-1, IL-1α, IL-6, and IL-9 contributing to a pro-inflammatory response [178,179] and stimulating angiogenesis [180].

Oxidative stress also indirectly influences AGE formation through both oxidation of lipids and sugars, thereby accelerating the rate of protein glycation and further contributing to ROS and reactive nitrogen species formation amplifying the cycle of cellular damage [181,182]. RAGEs are upregulated in Müller cells and RGCs [183] suggesting these cells are more prone to AGE/RAGE pathway signaling in glaucoma and DR, potentially also in AMD.

Spermine/spermidine, polyamines, are naturally occurring free radical scavengers with levels that decrease with aging, but supplementing either spermine, or its less potent spermidine derivative, either extra- or intracellularly has been shown to improve the lifespan of multiple cell models (e.g., yeast, Drosophila, human immune, and mouse RGCs) [48,184]. One possible mechanism of action of the spermine family of molecules is their ability to inhibit the activation of the MAPK pathway, mainly through activated ASK-1 and p38 proteins, by sequestering singlet oxygen and reactive species, e.g., ROS and reactive nitrogen species [48,185,186].

Increasing the expression of endogenous antioxidant genes is a clear way to attenuate an elevated level of oxidative stress by increasing the cell’s capacity to handle reactive species. Promising results have been reported when human extracellular SOD and catalase were transgenically expressed in eyes of DBA/1J mice, in which experimental autoimmune encephalomyelitis was induced subsequently [187]. Transgenic extracellular SOD and catalase expression limited RGC loss to 9% of normal optic nerve controls [187]. Viral delivery of a mitochondrial hydroperoxidase, peroxiredoxin 3 and a transcription factor regulating a cascade of “detoxifying” genes and enzymes, Nrf2, has also been shown to be effective at reducing oxidative stress in motor neurons of amyotrophic lateral sclerosis in vitro and in vivo models [188].

Interfering with RNA sequences have been an effective tool to advance basic research and with advancements in siRNA delivery, these molecules have been a target for drug development. Martínez and colleagues use a selective siRNA sequence (SYL040012) to target a specific β-receptors effectively decreasing IOP by ~20% after a mere 92 h after ocular administration [189]. By selecting for specific β-receptors using siRNA, the therapeutic strategy would limit the side effects associated with a broad inhibition of β-receptors, e.g., depression, impaired neuromuscular transmission, and sexual dysfunction. Wang et al. used an in vitro model utilizing human retinal microvascular endothelial cells to demonstrate the effectiveness of NADPH oxidase (i.e., Nox4) siRNA to alleviate VEGF-induced ROS production [190]. It remains interesting to note that by applying the approach of localized delivery of siRNA molecules, outlined by Martínez’ group [189], to retina tissue and the positive results obtained by Wang et al. [190] a potentially impactful therapeutic approach could be obtained.

### 4.2. Drug Development Efforts Targeting ROS: Small Molecule Strategies

#### 4.2.1. Glaucoma

As mentioned previously, traditional glaucoma therapy has focused on decreasing IOP although not all patients with glaucoma have increased IOP and IOP elevation does not necessarily lead to development of glaucoma [25,26]. Suppressing the effects and preventing the production of ROS, significant contributors to disease [22,23,24,25,27,28], via use of small molecules should thus be investigated as therapeutic options for glaucoma.

As alluded to previously, mitochondrial dysfunction is involved in neuronal cell death and thus represents a target for treatment of chronic neurodegenerative disease of the retina, including glaucoma. Although a poor ROS scavenger, estrogen may be implicated in the treatment of chronic neurodegenerative diseases due to its protective effects on mitochondria during times of stress. Indeed, 17β-estradiol inhibits ROS production, preserves ATP production, decreases cellular and mitochondrial calcium loading, and preserves mitochondrial membrane potential in SK-N-SH cells in the presence of an oxidative phosphorylation uncoupler. All of these events lead to significant neuroprotection [191]. Likewise, estrogen’s neuroprotective effects have translated to RGCs in an in vivo model of glaucoma [192]. Thus, estrogen seems to be a potential therapeutic option for preventing ROS-associated neurodegeneration characteristic of glaucoma.

Nrf2 is active in RGC neuroprotection via suppression of the harmful effects of ROS. Triterpenoids, activators of Nrf2, inhibit the production of ROS and degeneration of retinal capillary cells and RGCs in the presence of oxidative stress. These effects are not seen in Nrf2 knockout mice, indicating the specificity of the triterpenoids for Nrf2 and its importance in modulating oxidative stress to promote cell survival and function [193]. Nrf2 activation may represent an efficacious therapeutic approach in preventing the ROS-mediated effects of glaucoma and other neurodegenerative diseases.

ROS scavenging is a viable strategy for reducing the effects of ROS on RGCs. In addition to catalase, an H_2_O_2_ scavenger, manganese(III)tetrakis(1-methyl-4-pyridyl)porphyrin (MnTMPyP), Trolox, deferoxamine and SOD have been shown to reduce cell death caused by H_2_O_2_. This is particularly interesting, since none of the latter ROS scavengers scavenge H_2_O_2_. It is postulated that H_2_O_2_ is converted by reverse dismutation into superoxide or by the Fenton reaction into a hydroxyl radical. Alternatively, MnTMPyP may actually be a partial peroxidase, as it has been shown to scavenge H_2_O_2_ in cell-free assays. Trolox, deferoxamine, catalase, and SOD were also found to reduce RGC cell death caused by paraquat-generated superoxide anion. One explanation for these apparent superoxide scavenging-abilities is that superoxide is rapidly converted to H_2_O_2_ by superoxide dismutases. Deferoxamine, an iron chelator, would thus be able to prevent the conversion of H_2_O_2_ into a hydroxyl anion via inhibition of the Fenton reaction. Catalase and MnTMPyP were shown to decrease Fenton-mediated RGC death, likely by decreasing H_2_O_2_ substrate concentration and partial peroxidase activity, respectively. Trolox also decreased Fenton-mediated death, but to a lesser extent [193]. In light of this evidence, ROS scavengers seem to have therapeutic potential in treating neurodegenerative diseases. Moreover, it seems that certain scavengers, particularly MnTMPyP, catalase, and Trolox, have the ability to inhibit multiple ROS directly and indirectly, further implicating them as potential treatments.

#### 4.2.2. Diabetic Retinopathy

As discussed previously, as an antioxidant defense mechanism, cells may upregulate the activity of free radical ROS scavengers to minimize the harmful impact of oxidative stress. SERPINA3K is known to function as an endogenous antiangiogenic factor and is decreased in diabetic retinas. Intravitreal injection of SERPINA3K has been demonstrated to inhibit overexpression of VEGF and TNF-α and suppress ROS production in an oxygen-induced retinopathy model, likely at least in part due to concurrent upregulation of SOD and GSH. Together, these effects decrease ROS-induced retinal inflammation and neovascularization characteristic of DR [114]. Thus, future studies may investigate the role of retinal SERPINA3K level restoration/preservation in treating and preventing DR. Furthermore, other therapeutic modalities that increase ROS-scavenging enzyme levels in the retina may also be efficacious in management of DR.

Modulation of NADPH oxidase and Rac are important potential therapeutic approaches to treating the ROS-related dysfunction associated with DR. Minodronate, a recently developed bisphosphonate used for the treatment of osteoporosis inhibits retinal cell apoptosis and neuronal dysfunction in a rat model of DR via inhibition of NADPH oxidase-mediated oxidative stress generation [194]. Statins may also be implicated in decreasing the activity of NADPH oxidase in DR. Lovastatin prevented high glucose-upregulated expression of Nox4 (a subunit of NADPH oxidase), ROS generation, and VEGF levels in retinas of a diabetic mouse model, similar to the effect of a traditional NADPH oxidase inhibitor, diphenyleneiodonium chloride [195]. Thus, inhibition of NADPH oxidase may represent a potential therapeutic option in effectively decreasing ROS production to treat and prevent DR.

DR is characterized by increased vascular permeability propagated at least in part by leukostasis and increased leukocyte vascular adherence mediated by ICAM-1 upregulation. The p38 MAPK pathway plays a significant role in potentiating inflammatory responses (including upregulation of heat shock proteins, NADPH oxidase, etc.) and has been implicated in chronic neurodegenerative diseases of the retina. Blockage of this pathway via administration of PHA666859 (Pfizer, New York, NY, USA) has been shown to prevent diabetes-induced increases in superoxide, NO, cyclooxygenase-2, leukostasis, capillary degeneration, and pericyte death in diabetic rat retinas [196]. Thus, inhibition of the p38 MAPK pathway may be a worthwhile approach to prevent the inflammatory effects of ROS production associated with DR.

Heat shock protein 27 (Hsp27) lies downstream of and is phosphorylated by p38 MAPK. Phosphorylation of Hsp27 decreases its ability to defend against oxidative stress by binding to cytochrome c, activating Akt, and inhibiting caspase-3. Inhibition of the aforementioned p38 MAPK decreases Hsp27 phosphorylation, leading to inhibition of the development of vascular disease, likely contributing to the beneficial effects seen with PHA666859 administration [196]. Inflammatory cytokines and hyperglycemia appear to downregulate Hsp27, induce formation of ROS and stimulate apoptosis of human retinal endothelial cells. 1-methyl tryptophan has been shown to restore Hsp27 to control levels and inhibit the production of ROS. Tempol has also been shown to prevent cytokine-induced downregulation of Hsp27 in human retinal endothelial cells [197].

Moreover, as mentioned above, caspase cleavage of focal adhesion kinase (FAK) may be a critical event in ROS-induced epithelial cell apoptosis, perpetuating cell death. FAK cleavage and the corresponding apoptosis are almost completely prevented by administration of NAC [114]. Thus, ROS-mediated activation of caspases and their downstream activities may be worthy targets in the treatment of DR.

#### 4.2.3. Age-Related Macular Degeneration

The contributions of ROS to the development and progression of AMD seem to be diverse and significant. As a progressive, age-related disease comprising many cases of blindness among adults, effort must be directed toward preventing the manifestation of AMD. As significant contributors to disease, ameliorating the production and effects of ROS may be efficacious in doing so.

Endogenous regulation of redox state is important in the prevention of ROS-mediated damage associated with AMD. Apurinic endonuclease 1 plays a significant role in regulating redox state by facilitating the action of stress-responsive transcription factors, including NF-κB, activator protein 1, and HIF-1α. E3330, a small molecule inhibitor of apurinic endonuclease 1 has been shown to decrease retinochoroidal angiogenesis and reduce neuronal loss. E3330 also has RPE-protective effects associated with downregulation of intracellular ROS, suppression of nuclear accumulation of NF-κB, and inhibition of secretion of monocyte chemoattractant protein-1. E3330 was also shown to downregulate stress-responsive transcription factors (including those above) and reduce the progression of laser-induced CNV in vivo [137]. Thus, apurinic endonuclease 1 inhibition in the management of AMD may be worthy of further investigation.

Iron chelation may also play a potential therapeutic role in preventing the ROS-mediated manifestations of AMD. An effective chelator should ideally act only on that substance that is producing dysfunction within cells or tissues. This is particularly important and challenging for iron chelators due to iron’s abundance throughout the body and importance in normal physiology. Isonicotinic acid [2-(4,4,5,5-tetramethyl-[1,3,2]dioxaborolan-2-yl)-benzylidene]-hydrazide (BSIH) is a prochelator that is converted by H_2_O_2_ into salicylaldehyde isonicotinoyl (SIH). SIH then chelates iron that normally catalyzes hydroxyl radical generation. BSIH has been demonstrated to protect a model of RPE cells against H_2_O_2_-induced death and has proven to be stable and nontoxic to RPE cells [198]. Thus, prochelators, including BSIH, may represent an efficacious, targeted approach to mitigate the harmful effects of iron, a common catalyst for ROS production in the retina.

In the presence of TNF-α treatment, lycopene has also been shown to abolish NF-κB activation and ROS production in RPE cells, while increasing Nrf2 and GSH levels and decreasing ICAM-1 expression. The inhibition of NF-κB by lycopene is believed to be due in large part to Nrf2-induced antioxidant activity [199]. Thus, Nrf2 activation and/or NF-κB inhibition may be efficacious targets in preventing the ROS-induced manifestations of AMD.

Stimulation and downstream activity of caspases by ROS may also be targets for AMD therapy. Cleavage of FAK by caspases seems to be a critical event in ROS-induced epithelial cell apoptosis, accelerating cell death. FAK cleavage and apoptosis are almost completely prevented by the administration of NAC [200]. Thus, FAK cleavage and other ROS-induced pro-apoptotic events may be targets for AMD therapy.

### 4.3. Drug Development Efforts Targeting ROS: Biologicals

#### 4.3.1. Diabetic Retinopathy

As illustrated previously, angiogenesis is the distinguishing characteristic of proliferative DR and is at least partly regulated by ROS. Current therapy includes peripheral retinal photocoagulation, surgery, and anti-VEGF agents. Anti-VEGF agents have proven efficacy against angiogenesis but their function on RPE cell function and viability is less well known. Indeed, VEGF-A provides autocrine protection against oxidative stress in vascular endothelial and RPE cells, thus warranting investigation of the non-angiogenic effects of anti-VEGF agents. Recent evidence has shown that bevacizumab and aflibercept, anti-VEGF agents commonly used for treatment of retinal disorders associated with neovascularization including DR and AMD, have almost no toxic effect on RPE cells, ganglion cells, neuroretinal cells, and choroidal endothelial cells under normal conditions in vitro [91,93]. CXC chemokine platelet factor-4 (PF-4/CXCL4) is a chemokine ligand released from platelet granules upon activation and has been shown to inhibit human retinal microvascular endothelial cell signal transduction and migration induced by VEGF by binding to VEGF, disrupting VEGF-VEGFR binding and disrupting the intracellular cascade induced by VEGF. Patients with proliferative DR manifest increased levels of PF-4/CXCL4 in their vitreous fluid. Treatment with PF-4var/CXCL4L1 normalizes diabetes-induced VEGF and HIF-1α overexpression and correspondingly decreases human retinal microvascular endothelial cell permeability and RAGE and caspase-3 expression. PF-4var/CXCL4L1 is as effective as bevacizumab in decreasing BRB breakdown and vascular leakage [94], and thus may represent an alternative method of anti-VEGF treatment.

PEDF, the most important inhibitor of angiogenesis in the eye, may also be implicated as a therapeutic option in DR. Among other benefits, PEDF has been shown to suppress AGE receptor expression, ROS production, and AGE-induced IL-6 expression, monocyte chemoattractant protein-1 production, retinal vascular permeability and angiogenesis, platelet activation and aggregation, VEGF expression, and leukostasis [89,125,201,202,203,204,205]. PEDF administration also prevents the early neuronal derangements characteristic of DR, including decreased electroretinogram amplitude and increased glial fibrillary acidic protein levels. These effects are likely due to its inhibition of p22phox expression, a component of AGE-induced NADPH oxidase [206].

#### 4.3.2. Age-Related Macular Degeneration

RPE is an important source of VEGF and secretion of VEGF from RPE cells is a characteristic of tissues manifesting CNV. Oxidative stress stimulates the release of VEGF and thus plays a significant role in the pathogenesis of AMD, particularly the advanced dry and exudative forms [93]. Bevacizumab, ranibizumab, and aflibercept, commonly used anti-VEGF agents, have extra-angiogenic effects in that they have been shown to prevent acrolein-mediated death in RPE cells. Acrolein is a component of cigarette smoke and a product of lipid peroxidation that promotes mitochondrial damage-induced ROS production that is reversed by long-term treatment with the aforementioned anti-VEGF agents. Cell injury induced by acrolein seems to be mediated by MAPK pathway stimulation. Pretreatment with the aforementioned anti-VEGF agents has been shown to inhibit acrolein-induced redox-sensitive p38 and JNK activation. Furthermore, administration of all three of these agents enhances energy production and oxidative phosphorylation. Together, these results suggest that these agents mediate cellular protection by preventing activation of the MAPK pathways in RPE cells. Thus, by binding VEGF, these agents, particularly ranibizumab and aflibercept, prevent oxidative stress-mediated damage in RPE cells and the associated downstream events, including angiogenesis [207].

Importantly, however, it should be noted that administration of bevacizumab, but not ranibizumab or aflibercept, decreases RPE phagocytic activity, an important function of RPE cells in degrading outer segment photoreceptors. This may be explained by the fact that bevacizumab decreases mitochondrial bioenergetics after short-term exposure. Accordingly, bevacizumab provides no protection against mitochondrial damage induced by acrolein. On the other hand, aflibercept only promotes an early increase in mitochondrial bioenergetics but may protect against acrolein-induced RPE mitochondrial damage to a degree similar to that of ranibizumab, which promotes a long-term increase in mitochondrial bioenergetics. Interestingly, only long-term treatment with these three agents prevented acrolein-induced ROS overproduction and MAPK signaling [207]. Furthermore, aflibercept and bevacizumab administration has actually been shown to increase intracellular ROS and decrease intracellular VEGF, protein synthesis, and cell survival in RPE exposed to oxidative stress [91]. Furthermore, bevacizumab has been shown to decrease Bcl-2 expression in a dose-dependent fashion without affecting rates of apoptosis in the presence of low levels of oxidative stress. Bevacizumab increased RPE cell apoptosis in the presence of moderate to high oxidative stress but only when administered in doses higher than those used clinically. However, the decrease in Bcl-2 expression even in the presence of clinically applicable doses and low levels of oxidative stress warrants caution and close monitoring when using bevacizumab [93]. One method that may be used to dampen the potential harmful of effects bevacizumab is co-administration with an Fc receptor inhibitor. Indeed, incubation of RPE cells with an Fc receptor inhibitor suppressed the uptake of bevacizumab (but not aflibercept) into RPE cells, increased intracellular production of VEGF-A, decreased intracellular ROS, and enhanced RPE cell survival and proliferation under oxidative stress when compared with RPE cells exposed to only bevacizumab [91].

## Abbreviations

ROS	Reactive oxygen species
NADPH	Nicotinamide adenine dinucleotide phosphate
RPE	Retinal pigment epithelium
DNA	Deoxyribonucleic acid
RNA	Ribonucleic acid
IOP	Intraocular pressure
RGC	Retinal ganglion cell
TM	Trabecular meshwork
NMDA	*N*-methyl-D-aspartate
NO	Nitric oxide
SkQ1	10-(6′-pastioquinonyl) decyltriphenylphosphonium
ATP	Adenosine triphosphate
TNF-α	Tumor necrosis factor-alpha
H_2_O_2_	Hydrogen peroxide
SOD	Superoxide dismutase
HO	Heme oxygenase
GSH	Glutathione
Nrf2	Nuclear factor erythroid-derived 2
ASK1	Apoptosis signal-regulating kinase 1
NAC	N-acetylcysteine
Dock3	Dedicator of cytokinesis 3
DR	Diabetic retinopathy
PKC	Protein kinase C
AGE	Advanced glycation end product
RAGE	Receptor for advanced glycation end products
PEDF	Pigment epithelium-derived growth factor
mRNA	Messenger RNA
PI3K	Phosphoinositide 3-kinase
Akt	Protein kinase B
VEGF	Vascular endothelial growth factor
HIF	Hypoxia-inducible factor
IL	Interleukin
LDL	Low-density lipoprotein
CoCl_2_	Cobalt chloride
NADH	Nicotinamide adenine dinucleotide
NFAT	Nuclear factor of activated T cells
BRB	Blood-retinal barrier
MMP	Matrix metalloproteinase
ICAM-1	Intercellular adhesion molecule 1
siRNA	Small interfering RNA
AMD	Age-related macular degeneration
CNV	Choroidal neovascularization
Bcl-2	B-cell lymphoma 2
MAPK	Mitogen-activated protein kinase
JNK	Jun NH_2_-terminal kinase
MnTMPyP	Manganese (III) tetrakis(1-methyl-4-pyridyl)porphyrin
Hsp27	Heat shock protein 27
FAK	Focal adhesion kinase
BSIH	Isonicotinic acid [2-(4,4,5,5-tetramethyl-[1,3,2]dioxaborolan-2-yl)-benzylidene]-Hydrazide
SIH	Salicylaldehyde isonicotinoyl
PF-4/CXCL4	Platelet factor-4
